# Increased First Pass Success with C-MAC Videolaryngoscopy in Prehospital Endotracheal Intubation—A Randomized Controlled Trial

**DOI:** 10.3390/jcm9092719

**Published:** 2020-08-22

**Authors:** Christian Macke, Felix Gralla, Marcel Winkelmann, Jan-Dierk Clausen, Marco Haertle, Christian Krettek, Mohamed Omar

**Affiliations:** Trauma Department, Hannover Medical School, Carl-Neuberg-Strasse 1, 30625 Hanover, Germany; felix.gralla@st.ovgu.de (F.G.); winkelmann.marcel@mh-hannover.de (M.W.); Clausen.Jan-Dierk@mh-hannover.de (J.-D.C.); haertle.marco@mh-hannover.de (M.H.); krettek.christian@mh-hannover.de (C.K.); omar.mohamed@mh-hannover.de (M.O.)

**Keywords:** airway management, intubation, laryngoscopy, video, prehospital care, rescue helicopter, air ambulance

## Abstract

Endotracheal intubation (ETI) with direct view laryngoscopy (DL) is the gold standard for airway management. Videolaryngoscopy (VL) can improve glottis visualization, thus facilitating ETI. The aim of this monocentric, randomized, prospective study on a physician staffed German air ambulance is to compare DL and VL for ETI in terms of number of attempts and time as well as visualization of the glottis in a prehospital setting in a physician-based rescue system in adult patients. A power analysis was performed à priori. We used consecutive on-scene randomization with a sealed envelope system for the DL and VL-group. Successful ETI with first pass success was significantly more frequent with VL than DL and three seconds faster. The percentage of glottis opening and the Cormack & Lehane classification were significantly better with VL than DL. Regarding improved first pass success in ETI with the VL, we would recommend the use of VL for prehospital airway management in physician-based rescue systems.

## 1. Introduction

A difficult airway with a “cannot intubate—cannot ventilate” situation is a potentially fatal issue and a challenge for every physician [1]. Up to date, there is no simple reliable test for prediction of a difficult airway, therefore it is often impossible to perform the available tests in an emergency situation because of the missing opportunity for a structured evaluation of these factors.

In a physician based rescue system the gold standard in airway management should be endotracheal intubation (ETI). However, even in the routine elective surgery situation Adnet and colleagues reported a high rate of minor difficulties in ETI (37%) in a consecutive trial [2]. Additionally, Timmermann et al. could demonstrate a failure rate of right bronchus or esophageal intubation from emergency medical services in Germany of 10% and 6% in 2006 [3].

The question remains: How can we perform a safe ETI in the prehospital setting? A possible tool to manage the airway problem and establish a secure airway is the videolaryngoscope (VL) [4,5].

Loughan and co-workers found no significant difference for VL and direct view laryngoscopy (DL) in elective surgery [6]. Otherwise, a Cochrane review with 7044 participants undergoing elective surgery found moderate evidence for a reduction of failed intubation and laryngeal/airway trauma for VL and improved laryngeal view [7]. However, there was no evidence for a reduction in first pass success. In contrast, Pieters and colleagues showed in a meta-analysis of 1329 elective patients with known difficult airways a significant improvement even for the experienced anesthetist for first-pass success, laryngeal view, and a reduction of mucosal trauma [8]. Unfortunately, nearly all of these studies are performed as in-hospital studies with known sobriety anesthesia, complete medical history, and known risk factors.

In the past years some studies for prehospital videolaryngoscopy were published [9,10,11]. However, the results are quite distributed, ranging from only 48% first pass success rate up to 86% with different videolaryngoscopes. However, all of these studies are performed in air ambulance settings with experienced anesthesiologists as prehospital care physicians. They are on the one hand not representative for the common prehospital physician in Germany [12]. On the other hand, anesthetists are highly trained in direct laryngoscopy and therefore tend to have a lack of motivation to use this device as a new standard [13,14].

Therefore, the aim of this study was a comparison of DL and VL with regard to first-pass success and time of attempts as well as glottis visualization in a prehospital setting in a physician-based rescue system in adult patients. We hypothesized that VL will lead to a higher first pass success rate and a better visibility of the glottis for non-anesthetist prehospital physicians.

## 2. Experimental Section

Ethical approval was obtained by the ethical board of Hannover Medical School with the registration number 2016–7268. In accordance with the requirements of the ethical approval, informed consent was obtained after recovery of the patient. In case of death or permanent disability consent was obtained from relatives.

Prior to study, a power analysis with sample size calculation was performed for the first pass success rate. For 80% quality with a significance level of *p* < 0.05 the sample size was set to 76 patients per group, resulting in a total patient number of 152, assuming a first pass success rate of 80% in the DL [15] and 95% in the VL group [16].

**Figure 1:**
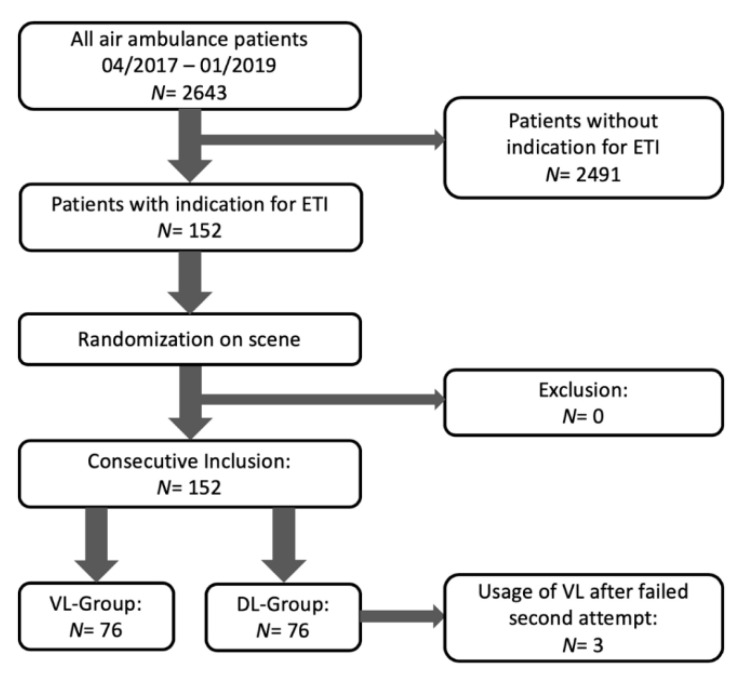
Flow-Chart of patient enrollment.

We performed a prospective, consecutive, and randomized enrolment of all adult patients with indication for intubation on the air ambulance from 04/2017–01/2019 (Figure 1 Flow Chart).

### 2.1. Primary Outcome

First pass success rate in comparison of DL vs. VL.

### 2.2. Secondary Outcome

Visualization of the glottis in comparison of DL vs. VL.

Comparison of success rate of less experienced physicians with <100 intubations (LEP) vs. experienced physicians with >100 intubations (EP).

Inclusion criteria: Age ≥ 18 years; Necessity of endotracheal intubation.

Exclusion criteria: Age < 18 years.

### 2.3. Intubation and Staff

Intubations were performed with rapid-sequence induction in all patients >GCS 3 in a team approach of a physician with the help of a HEMS paramedic. In patients with GCS 3 the intubation was conducted without drugs. The treating physician had free choice of induction drug, but was instructed to use a relaxant according to our standard operating procedures.

#### 2.3.1. Physician

In contrast to most other German air ambulances all our physicians are experienced trauma surgeons with an additional qualification for prehospital care. The experienced group had an experience > 100 intubations (EP) prior to this study, the less experienced group < 100 intubations (LEP) in total.

#### 2.3.2. HEMS Paramedic

The Helicopter Emergency Medical Service paramedics are specially trained and are qualified to prepare ETI as well as support the physician during the intubation procedure. In this study the HEMS paramedic conducted the documentation, drug administration, and time measurement.

### 2.4. Randomization

Immediately after the decision for endotracheal intubation the HEMS paramedic pulled a sealed, opaque envelope with the randomized method and prepared the equipment for intubation. The physician only knew the method right before the intubation. The envelopes were prepared with a consecutive numbered standardized protocol (see measured parameters) in advance by author FG, who was not involved in the treatment of the patients.

### 2.5. Airway Management Problems

In case of ETI problems with DL the physician was allowed to change the method to VL anytime but had to switch to VL after the second fail with DL. Other back-up systems were also available (laryngeal tube, bag-mask-ventilation, surgical airway).

### 2.6. Laryngoscope

The standard laryngoscope was a Vital View II LED from GE, Boston, Massachusetts, USA with single use blades, whereas the videolaryngoscope was the C-MAC^®^ PM from Storz Medical, Tuttlingen, Germany with multi-use blades (Macintosh II-IV, D-Blade). The videolaryngoscope has been available on the air ambulance since 2016. Moreover, every physician had a briefing into the usage prior to the study and had to conduct at least ten intubations on an airway trainer.

### 2.7. Measured Parameters

All of these parameters were documented on a standardized protocol after performing the intubation:

- Assumed intubation problems (facial trauma, limited mouth opening (<2.5 cm), no neck, rigid collar during intubation, fluid in pharynx (vomit/blood)).
- Indication (resuscitation/CPR (cardiopulmonary resuscitation), trauma-resuscitation/trauma-CPR, musculoskeletal trauma, neurologic, burns, pulmonary, drowning).
- Site (floor, ambulance stretcher, sitting position, inside helicopter, other).
- Lighting conditions (poor, good, too bright).
- Necessary or applied relaxation with induction.
- Oxygen saturation before, during and after intubation.
- Best sight of glottis with percentage of glottis opening (POGO-Score) and Cormack-Lehane Score (CL) I–IV.
- Time for intubation measured by the HEMS paramedic with a stopwatch from taking off the mask to either tube blocking and detectable end-tidal CO_2_ or putting back the mask.
- In case of videolaryngoscopy usage: whether the monitor or direct laryngoscopy was used. In all cases the monitor was used.
- Number of attempts with video- or direct view laryngoscopy.
- Blade size and type (Macintosh II-IV, D-Blade).
- Correctness of intubation proofed by auscultation and capnography.
- Necessity of alternative airway management.

### 2.8. Statistical Analysis

Statistical analysis was performed with SPSS 25 (IBM). For dichotomous variables the Fisher’s-Exact-Test was used, and for mean variables the Mann-Whitney-U-Test after checking for normal distribution. Significance level was set to *p* < 0.05.

## 3. Results

### 3.1. General Patient Data

Median age of the patients was 68 years (Q1:Q3; 55:78) and they were predominantly male (*n* = 113 (74.3%) vs. *n* = 39 (25.7%)). No alternative airway was necessary in any of the 152 cases. Thirty-four patients (22.4%) died at the scene of ETI, all other patients were brought to hospital alive (*n* = 118 (77.6%)). All patients, including the deceased, had a documented detectable end-tidal CO_2_. There were no differences in basic parameters in both groups. The site of intubation was as follows: stretcher of an ambulance car (*n* = 79 (52.0%)), ground (*n* = 72 (47.4%)), sitting position in a car due to entrapment (*n* = 1 (0.7%)). This was handled without difficulties in the first attempt with VL. For distribution of indications for ETI see also Table 1.

There were no differences in the DL and VL group in view of lighting conditions, medical indication, intubation site, as well as necessary or applied relaxation. All patients with GCS > 3 had a relaxant administered with induction. Possible intubation problems—defined as facial trauma, limited mouth opening (<2.5 cm), no neck, rigid collar during intubation, fluid in pharynx (vomit/blood)—were found in 50% of the patients in each group (Table 2). There were no significant differences regarding the above mentioned assumed possible intubation problems between DL and VL group.

We aimed to evaluate the oxygen saturation before, during, and after intubation. Unfortunately, only in *n* = 71 (46.7%) cases these parameters were documented sufficiently. Nearly all cases were unproblematic intubations in the first attempt with a desaturation no more than ten per cent. Especially in CPR situations the values were poorly documented or not measurable.

### 3.2. Experienced vs. Less Experienced Physicians

No difference was found between the group of experienced physicians and less experienced physicians for all parameters. The groups were distributed equally with 48% (47/98) VL usage for the EP and 54% (29/54) VL (*p* = 0.6) usage for the LEP.

### 3.3. First Pass Success Rate

No alternative airway management had to be used in any of the 152 patients. Table 3. Displays the success rate in relation to the number of attempts. VL resulted in 95% successful ETI at the first attempt compared to 79% with DL. All of the four unsuccessful first attempts with VL were interrupted attempts because of massive aspiration and/or pharyngeal bleeding before placing the tube, and subsequent necessity for suction, whereas only three of the 16 s attempts with DL group were related to this issue. The others were due to visibility problems. All aspirations or bleedings, except for one aspiration in group DL and one in group VL after induction respectively, occurred before the first intubation attempt and were not due to induction or intubation. Moreover, 100% (76/76) in the VL group had a correctly placed tube after two attempts, whereas only 96% (73/76) in the DL group were placed successfully at the second attempt.

Of the three patients with a second failed attempt with DL two could be intubated successfully (67%) with VL in the first attempt. Only one patient with a Cormack-Lehane Score of IV needed a second VL attempt.

The number needed to treat (NNT) for the first pass success with VL was 6.3, and the absolute risk reduction was 0.16.

### 3.4. ETI Time and Glottic View

Median ETI time at the first attempt of VL and DL was 15.5 s (Q1:Q3; 10:20) and 18.5 s (Q1:Q3; 12.5:24.5) (*p* = 0.01). See Table 4. For all attempts.

Overall, VL leads to a significantly better glottic view in the first attempt (Table 5).

## 4. Discussion

This prospective, consecutive and randomized study compared the success rate of the video-laryngoscopic ETI with the conventional ETI in a preclinical setting of a German air ambulance.

The primary goal was to address the question whether the first pass success, meaning an successful ETI in the first attempt, was higher in the VL group, as it is well-known that more than one attempt for ETI in an emergency situation is a significant predictor for adverse events [17].

In our study we demonstrated a first-pass success rate of 79% with DL and 95% with VL, which is comparable to the existing literature for DL but better for VL in the prehospital setting [10,11]. The first pass success rate for in-hospital emergency ETI ranges from 75% to 85% with DL [15,18,19], and up to 96% with VL [16,20,21]. Mackie and colleagues could demonstrate that first pass success could be significantly increased from 59.2 to 85.1% with the C-MAC video-laryngoscope in 163 emergency intubations by emergency registrars [22]. Moreover, the complication rate dropped from 28.9 to 16.1% in their study.

A possible reason for the higher success rates with VL might be improved glottis visibility, as it is long known for DL that a better view leads to higher success rates [23]. Piepho and colleagues demonstrated in 52 patients with a Cormack & Lehane grade III an improvement with VL in 94% of the patients [24] and Sulser and his co-workers reported a significantly better Cormack & Lehane grade for VL in a randomized trial in an emergency department [25], although they were not able to demonstrate a higher first-pass success rate as they had a first-pass success of almost 100% in both groups. One possible explanation for these findings could be that all of the intubations were performed by three very experienced anesthesiology consultants under favorable conditions in an emergency department. However, this is not the usual setting for out of hospital ETI and the majority of prehospital active emergency physicians in Germany are not that well experienced, since only about 25% are consultants [12].

We aimed to evaluate the desaturation during intubation as oxygen saturation is a crucial factor during intubation [26]. Bodily and colleagues found in their study of *n* = 265 rapid sequence inductions in an emergency department a desaturation in 35.5%, but had to exclude 99 patients from their study due to unavailable data, although they used electronic data acquisition [26]. Unfortunately, our documentation here is not adequate with data only recorded in 46.7% of cases. It should also be mentioned that all intubations were performed in a team approach with reduced resources. Especially in CPR situations with only a team of two persons the possibility for documentation is not always possible without endangering the patient. In our cohort more than half of the patients were patients with CPR/Trauma-CPR with an initial GCS of 3. Moreover, the time for intubation was quite short, so that desaturation was unlikely.

These points demonstrate the difficulty in transferring data from emergency in-hospital situations to the rough environment in the prehospital setting.

Furthermore, it should be mentioned that the type of videolaryngoscope could have an influence on first pass success, too. Ruetzler and colleagues found different success rates in a comparison of five different videolaryngoscopes in a training situation in which C-MAC performed very well in difficult airway situations [27]. Cavus et al. however, found the C-MAC only comparable to the A.P.Advance but better than the KingVision [11].

Thus, the results for videolaryngoscopy with 95% first pass success could be linked to the type of videolaryngoscope employed. Another possibility could be the profession of the physicians: all of the physicians are trauma surgeons with training in arthroscopy. They are used to perform complex triangulation on a 2D screen. Maybe this expertise helps in performing ETI with VL.

In this study there was no difference between well-experienced physicians with more than 100 ETIs and less-experienced physicians. Referring to Mackie and colleagues LEP probably were able to intubate with high success rates with VL [22] and the success rates with DL are comparable to other physician-based preclinical ETI rates with DL [19]. Because of the study design with focus on comparison of VL and DL, preplanned patient number in both groups for this particular question would be too small to answer it with high statistical power. On the other hand, we had no necessity for an alternative airway in any of the 152 patients, and even two out of three patients (67%) with two failed DL attempts could be intubated with VL at the first try.

Another finding of our study was the three-seconds-faster ETI with VL. The absolute time seems quite fast with 16 and 19 s, respectively. Different authors reported intubation times around 30 s [6,25]. However, it is difficult to compare the absolute time for intubation procedure as the definition of beginning and ending differs vastly. Moreover, we are convinced—in accordance with the literature—that time is not as crucial as the first-pass-success, as long as there is no desaturation with hypoxia during intubation [7,15].

### 4.1. Limitations, Strength, and Generalizability

There are limitations to this study. First, the study was only performed on one air ambulance in Germany with physicians experienced in prehospital care, which limits the results to physician-based prehospital care with the use of rapid-sequence induction. Moreover, all of the physicians are trauma surgeons without any anesthesiological background, which limits the generalizability of this study.

On the other hand, this could be a strength as well. Only half of the physicians had performed more than 100 intubations in their medical career. However, the results of this study are comparable to other anesthesiological studies where only experienced anesthesiologists performed emergency intubations, but better regarding videolaryngoscopy [5,9,10]. Furthermore, our group of physicians is very homogenous with relatively low experience in ETI from 50 to 300 in total. This represents the German reality in prehospital care even better than well-experienced anesthesiologic consultants would do [12]. With regard to the relatively low experience in ETI of our physicians and the high rate of patients without the need for rapid-sequence-induction, this study may be applicable for paramedics as well.

A further strength of this prehospital study with prospective randomization is the consecutive inclusion of the participants without any loss of recruitment or exclusion of patients.

Moreover, we performed a prior power analysis and assumed a distribution of 80% (DL) and 95% (VL) first pass success for an 80% quality. It should be mentioned that we assumed the different values based on one review and meta-analysis of Park et al. [15] which deals with intubations in emergency departments. Furthermore, because of a lack of high-volume studies or wide distribution regarding the success rates of videolaryngoscopy in emergency situations, we used Aziz et al. [16] to estimate the success rate of videolaryngoscopy. This study is a retrospective in-hospital study. It could be debated whether this is transferrable to our study. Otherwise, the prior power analysis correlates very closely with our results.

### 4.2. Summary

We could demonstrate a significantly better first pass success, a better glottic view and a slightly faster ETI with VL. Since most of the prehospital active emergency physicians in Germany are not very experienced in ETI [12] and younger and less experienced physicians benefit most from VL [20,22], we would recommend videolaryngoscopy as primary device for ETI in prehospital care at least in a physician-based system.

## 5. Conclusions

In this prehospital randomized study comparing videolaryngoscopy and direct laryngoscopy we showed a significant advantage of videolaryngoscopy in view of success at the first attempt in prehospital endotracheal intubation. Therefore, we recommend videolaryngoscopy as the primary device for airway management with endotracheal intubation in the prehospital setting in a physician-based rescue system. Since the completion of this study we use videolaryngoscopy as the primary device in our own air ambulance.

## Figures and Tables

**Table 1:** Indication for endotracheal intubation (ETI) in the study population.

Indication	Number (*n* (%)) Total	Number (*n* (%)) VL	Number (*n* (%)) DL
Resuscitation/CPR	70 (46.1%)	29 (38.2%)	41 (53.9%)
Trauma-resuscitation/Trauma-CPR	14 (9.2%)	10 (13.2%)	4 (5.3%)
Musculoskeletal trauma	47 (30.9%)	27 (35.5%)	20 (26.3%)
Neurologic	11 (7.2%)	6 (7.9%)	5 (6.6%)
Burns	5 (3.3%)	1 (1.3%)	4 (5.3%)
Pulmonary	4 (2.6%)	2 (2.6%)	2 (2.6%)
Drowning	1 (0.7%)	1 (1.3%)	0 (0.0%)

CPR: cardiopulmonary resuscitation; VL: videolaryngoscopy; DL: direct view laryngoscopy.

**Table 2:** Comparison of videolaryngoscopy (VL) and laryngoscopy (DL) of possible intubation problems.

	VL (*n* (%; 95%-CI))	DL (*n* (%; 95%-CI))	*p*-Value
Number	76	76	-
Rigid collar during ETI	19 (25%; 15–35)	11 (14%; 6–23)	0.15
No neck patient	12 (16%; 7–24)	8 (11%; 3–18)	0.47
Mid facial trauma	10 (13%; 5–21)	8 (11%; 3–18)	0.80
Bleeding/aspiration	10 (13%; 5–21)	12 (16%; 7–24)	0.82
Limited mouth opening	6 (8%; 2–14)	3 (4%; −1–8)	0.49
Assumed intubation problems	42 (55%; 44–67)	39 (51%; 49–63)	0.75

**Table 3:** Number of attempts for VL and DL.

Attempts	VL (*n*, (%; 95%-CI))	DL (*n*, (%; 95%-CI))	*p*-Value
1st attempt successful	72 (95%; 90–100)	60 (79%; 70–88)	0.007
2nd attempt successful	4 (100%; 100)	13 (81%; 60–103)	1.0
3rd attempt successful	0 (0%)	2 (67%; −77–210)	not applicable
4th attempt successful	0 (0%)	1 (100%; 100)	not applicable

**Table 4:** Duration in relation to number of attempts for VL and DL in seconds (s).

Attempts	VL (s) as Median (Q1:Q3)	DL (s) as Median (Q1:Q3)	*p*-Value
1st attempt	15.5 (10:20)	18.5 (12.5:24.5)	0.01
2nd attempt	15 (9.0:25.0)	15 (10:20)	0.89
3rd attempt	n. a.	12 (10.5:18.5)	n. a.
4th attempt	n. a.	30	n. a.

**Table 5:** Visibility of glottic opening in the first and second attempt for VL and DL.

**Dependence on 1st Attempt**	**VL**	**DL**	***p*-Value**
POGO-Score (%), median (Q1:Q3)	100 (90:100)	65 (30:90)	<0.001
Cormack & Lehane, median (Q1:Q3)	1 (1:2)	2 (2:2)	<0.001
**Dependence on 2nd Attempt**	**VL**	**DL**	***p*-Value**
POGO-Score (%), median (Q1:Q3)	72.5 (51.25:90)	20 (0:62.5)	0.04
Cormack & Lehane, median (Q1:Q3)	2 (2:2)	2 (2:3.75)	0.29

POGO: percentage of glottis opening.
